# Creutzfeldt-Jakob disease presenting as psychiatric disorder: case presentation and systematic review

**DOI:** 10.3389/fneur.2024.1428021

**Published:** 2024-08-29

**Authors:** Brendan Huang, Neeva Shafiian, Paul Joseph Masi, Marc L. Gordon, Ana M. Franceschi, Luca Giliberto

**Affiliations:** ^1^Department of Neurology, Northwell, New Hyde Park, NY, United States; ^2^Zucker School of Medicine at Hofstra/Northwell, Hempstead, NY, United States; ^3^Department of Psychiatry, Northwell, New Hyde Park, NY, United States; ^4^Departments of Neurology and Psychiatry, Donald and Barbara Zucker School of Medicine at Hofstra/Northwell, Hempstead, NY, United States; ^5^Feinstein Institute for Medical Research, Northwell Health, Manhasset, NY, United States; ^6^Department of Radiology, Northwell, New Hyde Park, NY, United States

**Keywords:** Creutzfeldt-Jakob disease, mania, psychosis, depression, systematic review

## Abstract

Creutzfeldt-Jakob disease (CJD) is a spongiform encephalopathy caused by misfolded human prion proteins (PrP)s. Due to variability in presentation, the diagnosis may be missed in lieu of various psychiatric disorders. Our study reports on a prototypical case and psychiatric mimic for CJD, and the workup used to establish the correct diagnosis. A 54-year-old male with a past medical history of traumatic brain injury and major depressive disorder presented with chest pain. During the hospital stay, he was found to be increasingly aggressive, and behaved out of character. Further review of clinical history revealed that the patient was diagnosed with cognitive impairment and depression one year prior. The patient was agitated, poorly redirectable, and had unstable gait on neurological examination. Magnetic resonance imaging (MRI) of the brain demonstrated restricted diffusion (DWI) along the parietooccipital and temporal regions (L > R) and in the subcortical structures, including the basal ganglia and thalami, with accompanying subtle fluid attenuation inversion recovery (FLAIR) hyperintense signal abnormality in these regions, deemed as artifactual at the time. Repeat MRI brain two months later demonstrated progression of the DWI signal with ADC correlate and FLAIR findings. Cerebrospinal fluid 14-3-3 and RT-QuIC samples were positive. Upon passing a few months later, brain autopsy and Western Blot confirmed the CJD diagnosis. Literature review was conducted on PubMed to identify CJD cases initially diagnosed as psychiatric disorder. Search terms included “CJD” or “Creutzfeldt-Jakob disease” with three common psychiatric diagnoses, “Depression,” “Psychosis,” and “Mania.” Positive EEG, MRI, PET, and CSF (including protein 14-3-3 and tau) findings for CJD were found in 66.7, 81.1, 50, and 72.7% of cases, respectively. Overall, CJD can present as a psychiatric mimic. In suspicious cases, EEG, imaging, and CSF studies should be promptly utilized to arrive at the correct diagnosis. Repeated MRI imaging is often required to help in the diagnostic process. Brain biopsy should be considered in selected cases.

## Introduction

Creutzfeldt-Jakob disease (CJD) is a type of spongiform neurodegenerative disease caused by misfolded human prion protein (PrP) that is rare, rapidly progressive, and invariably fatal (1). The disease has an equal prevalence across sexes, races, geography, and socio-economic status in the United States (2). Four forms of the disease exist: sporadic (85% of cases), familial, acquired, and variant CJD (1). The case report rate is 1 per million annually, with an average age of symptom onset of 61 (3, 4). Recent studies in the Japanese population have demonstrated an increase in age-adjusted mortality and incidence rates since 2005, disproportionally in older and female individuals (5, 6). This finding may reflect changing demographics versus improving detection methods through imaging and other diagnostic testing. The disease is characterized by a devastating loss of cognitive and motor function resulting in akinetic mutism, with the majority of patients passing away within a year of symptom onset (7, 8). Some cases demonstrated long asymptomatic incubation periods, allowing the possibility for transmission and posing a high cost burden and potential public health risk (3, 9). With the average mean time from symptom onset to diagnosis approximately 8 months, identifying patients at an early stage is critical and physicians should require a lower threshold for ordering additional diagnostic tests in suspected cases.

Brain biopsy is currently the gold standard for diagnosing CJD (10). However, less invasive tools may be employed in order to fit the diagnostic criteria of CJD. Imaging techniques, such as MRI and positron emission tomography (PET) scans are used to identify hallmark characteristics of CJD. Cerebrospinal fluid biomarkers, such as 14-3-3 and tau, are also typically elevated in patients with CJD, but are not specific. Since 2010, cerebrospinal fluid real-time quaking-induced conversion (RT-QuIC) has been a crucial diagnostic tool for identifying CJD, with high levels of specificity and sensitivity (11, 12). Once patients are diagnosed, treatment options are limited to palliative care interventions; thus, identifying CJD early allows for correct management, emotional and spiritual support for patients and families, with discussions of goals of care.

In its classic form, CJD has several identifiable features; clinical findings include myoclonus, pyramidal/extrapyramidal signs, and dementia. However, it has been reported that about 26% of cases initially present with nonspecific psychiatric symptoms such depression, mania, and psychosis, with 80% of patients experiencing psychiatric symptoms within the first 100 days (13). Such presenting symptoms often result in the misdiagnosis of a psychiatric mimicker, delaying CJD diagnosis and palliative treatment.

Here, we describe the case of a 54-year-old man who presented with symptoms, initially thought to be due to a psychiatric condition progressing from a traumatic brain injury, who was later found to have CJD. Afterwards, we present a systematic review of the literature on patients initially presenting with common psychiatric symptoms of depression, mania, and psychosis, who were later confirmed to have CJD. We analyze the diagnostic tools that were used to arrive at the correct diagnosis.

## Case presentation

**Figure 1:**
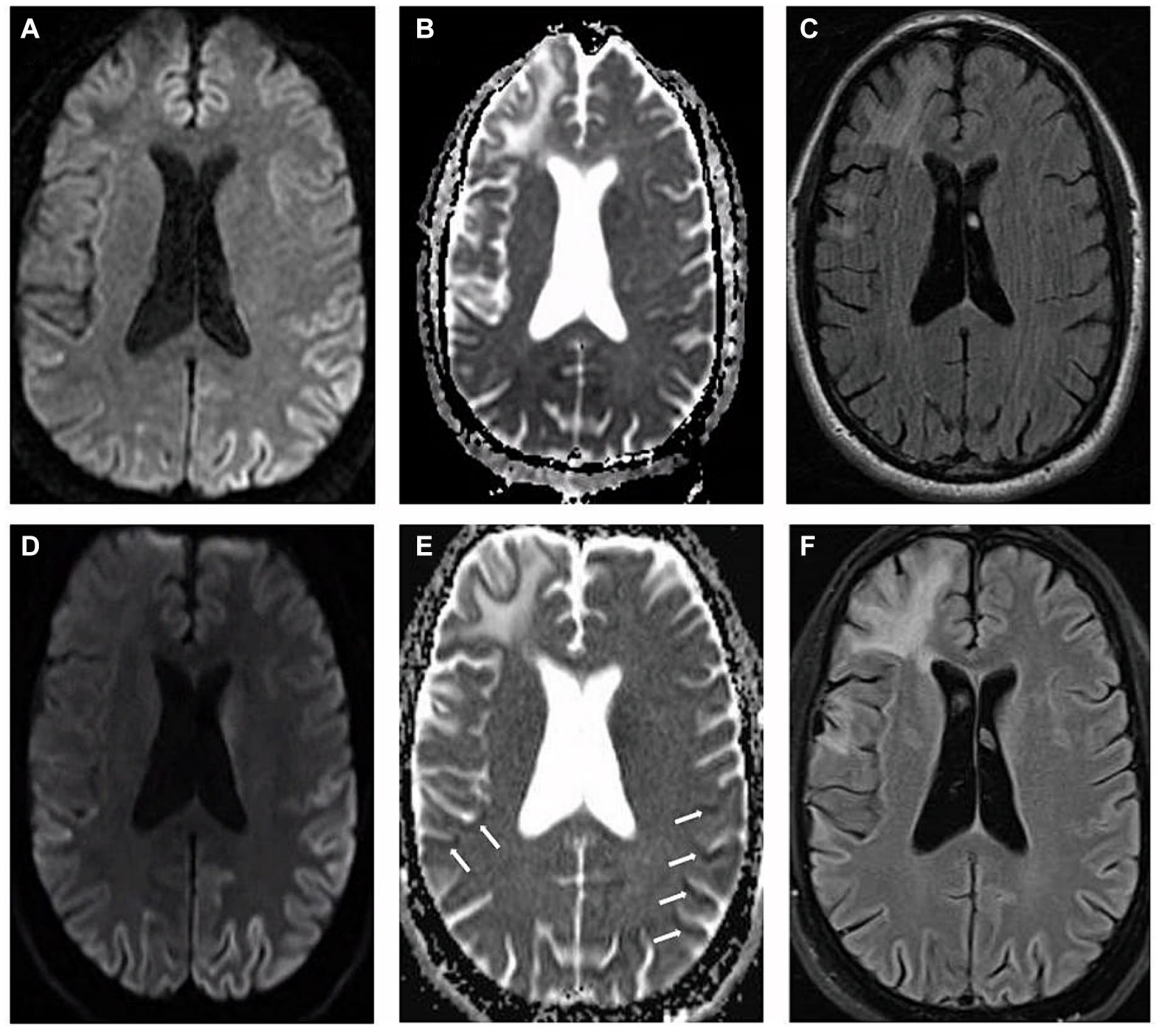
Magnetic resonance imaging: **(A–C)** represent first MRI scan in March, 2022 and **(D–F)** demonstrate second MRI scan acquired two months afterwards. **(A,D)** Axial diffusion weighted imaging **(B,E)** axial apparent diffusion coefficient imaging **(C,F)** axial T2 fluid attenuated inversion recovery imaging. White arrows indicate the locations of apparent diffusion coefficient correlate.

We present a case of a 54-year-old male who suffered from traumatic brain injury (TBI) after falling from a ladder at the age of 45 with seemingly no immediate sequelae. His past medical history also included hyperlipidemia and major depressive disorder. The patient was dismissed from his job as a plumber in late 2021, due to cognitive changes, inability to be redirected, an inability to identify and name everyday objects such as coffee cup and glasses leading to significant frustration; he was described as having “no filter” making inappropriate remarks towards others. He attempted to improve his medical condition by removing from stress and relocating to a different state, seeking therapy and treatment. However, by March, 2022, the patient was noted by family to have ever-worsening communication, despite ongoing speech therapy, and orientation, having being found lost while driving. An outpatient MRI brain acquired at that time demonstrated evidence of gliosis and encephalomalacia in the right fronto-temporal region in a pattern compatible with TBI sequelae, as well as suggestion of restricted diffusion throughout the cortex, most pronounced in the left parietal lobe (Figures 1A–C). It was assumed that the TBI was leading to progressive cognitive decline, including speech and memory deficits. At the time, the increased signal on diffusion weight imaging (DWI) with no apparent diffusion coefficient (ADC) correlate was interpreted as artifactual.

By early April 2022, the patient’s cognition continued to deteriorate, and the family decided to bring him back to his original state of residence for further treatment. On one occasion, he presented to the emergency room (ER) for agitation and was started on valproic acid for mood control, later reduced for agitation. In late April, he presented to the ER again for palpitations and chest pain. Electrocardiogram and troponin levels were normal. The patient was initially cleared for discharge but due to intense agitation with physical, and verbal aggression, was routed for inpatient psychiatry admission. While the patient was alert and oriented to person, place, and situation, the psychiatric interview was limited by expressive and, suspected, receptive aphasia with decreased productivity, increased latency, and impaired articulation. The patient was noted to have good hygiene and grooming. He had fair eye contact but poor relatedness to the interviewer. His impulse control, attention, memory, fund of knowledge, language, judgement, and insight were noted to be impaired.

The patient was eventually seen by inpatient neurologists in May 2022, due to continued cognitive decline. A full neurological examination revealed good behavioral control and fair eye contact. The patient’s speech was fluent, not pressured, but not understandable, characterized by predominantly semantic paraphasia. He did not follow commands for a full cranial nerve assessment, but extraocular movements were noted to be intact with no gross facial asymmetry. Motor testing was limited by the patient’s inability to completely follow commands but demonstrated normal bulk and tone, without focal motor deficits. He demonstrated normal motor praxis (basic organized movements, manipulations, “reaching for the door”) but had severe ideational and ideomotor apraxia which was compounded by a tendency to perseverate and imitate inappropriately, while demonstrating gestures irrelevant to the task, almost parasitic. He did not have myoclonus in the awake state. He exhibited hyperreflexia on all extremities with a positive glabellar reflex. He could walk unassisted but was noted to have hesitant and apraxic gait with a narrow base. The patient said that he could “feel his agitation coming and cannot control the feeling” and that these symptoms occurred at night requiring sedating medications. His sleep was described as “restless” with inconsistent right upper extremity focal myoclonus with sporadic intrusive event and episodes of apnea. After a thorough review of the first MRI, it was decided to repeat the scan during the patient’s current admission due to clinical suspicion of CJD. Imaging, again, demonstrated restricted diffusion along the parieto-occipital and temporal cortex with apparent diffusion coefficient (ADC) correlation, findings more pronounced on the left side, compatible with CJD (Figures 1D–F). Cerebrospinal fluid was drawn for RT-QuiC, 14-3-3, Phosphorylated-TAU, total-TAU, amyloid beta 42, cell count, glucose, lactate dehydrogenase, and protein levels.

During the same admission, patient was started on antipsychotics for severe and violent agitation. He was eventually transitioned to a combination of escitalopram 15 mg daily, quetiapine 300 mg nightly, sodium valproate 500 mg every 12 h, and melatonin 3 mg nightly with significant improvement in insomnia and agitation. With the decrease in agitation, he was noted to have some improvement in ataxia and apraxia. He was later discharged home from the inpatient psychiatry unit with recommendations for outpatient psychiatry follow up. Lumbar puncture demonstrated elevated 14-3-3 protein and the results were shared with the family with recommendations for hospice care, given high probability of CJD.

The patient returned to an outside hospital one month afterwards for further agitation refractory to antipsychotics. He eventually required inpatient hospice care and was started on a continuous infusion of sedatives such as hydromorphone, midazolam, and ketamine. The patient passed away on July 22, 2022 from cardiopulmonary failure. Positive RT-QuIC results arrived shortly after death, further confirming the diagnosis of CJD.

**Figure 2:**
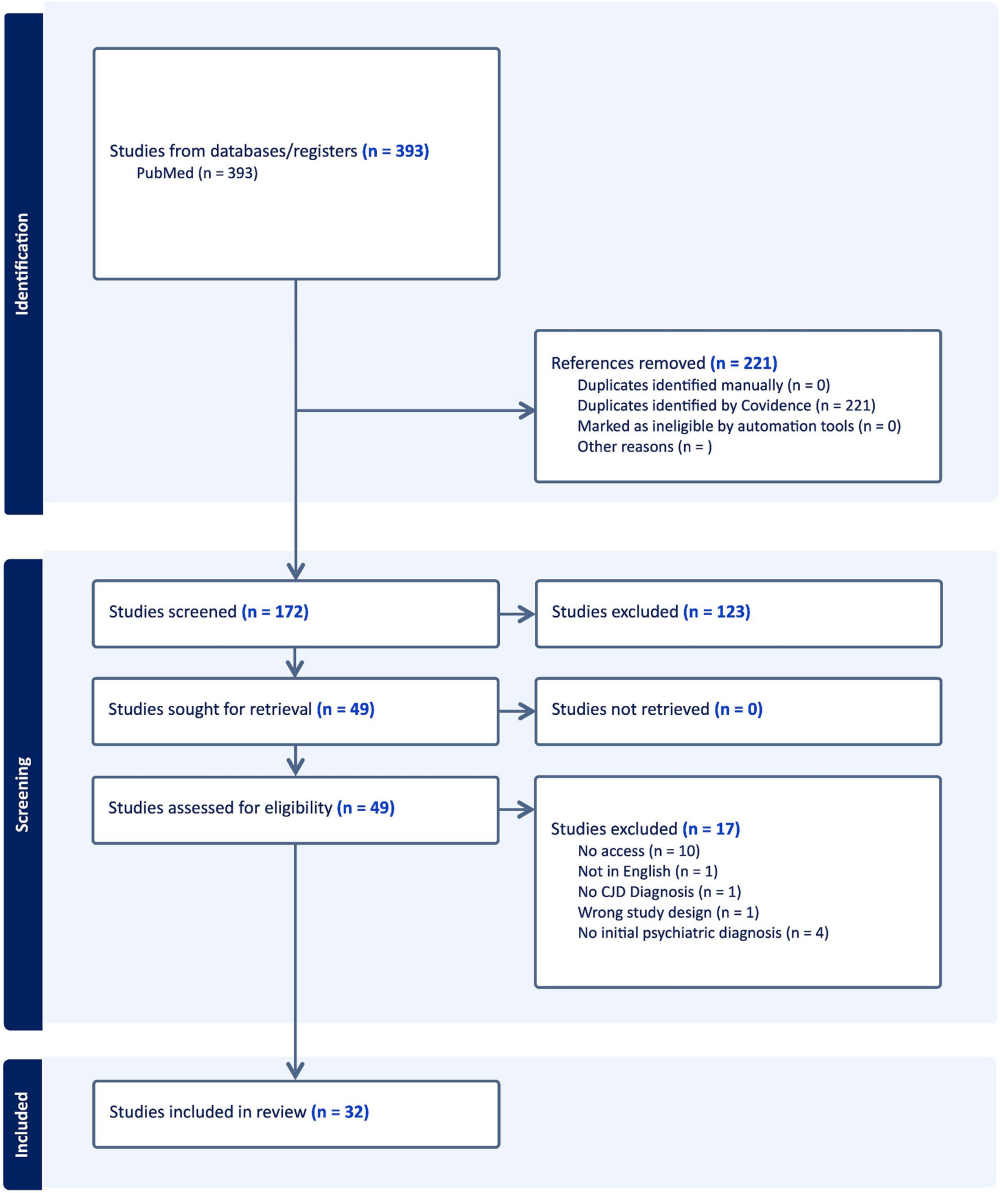
PRISMA schema: diagrammatic representation of literature search utilizing PubMed database.

**Figure 3:**
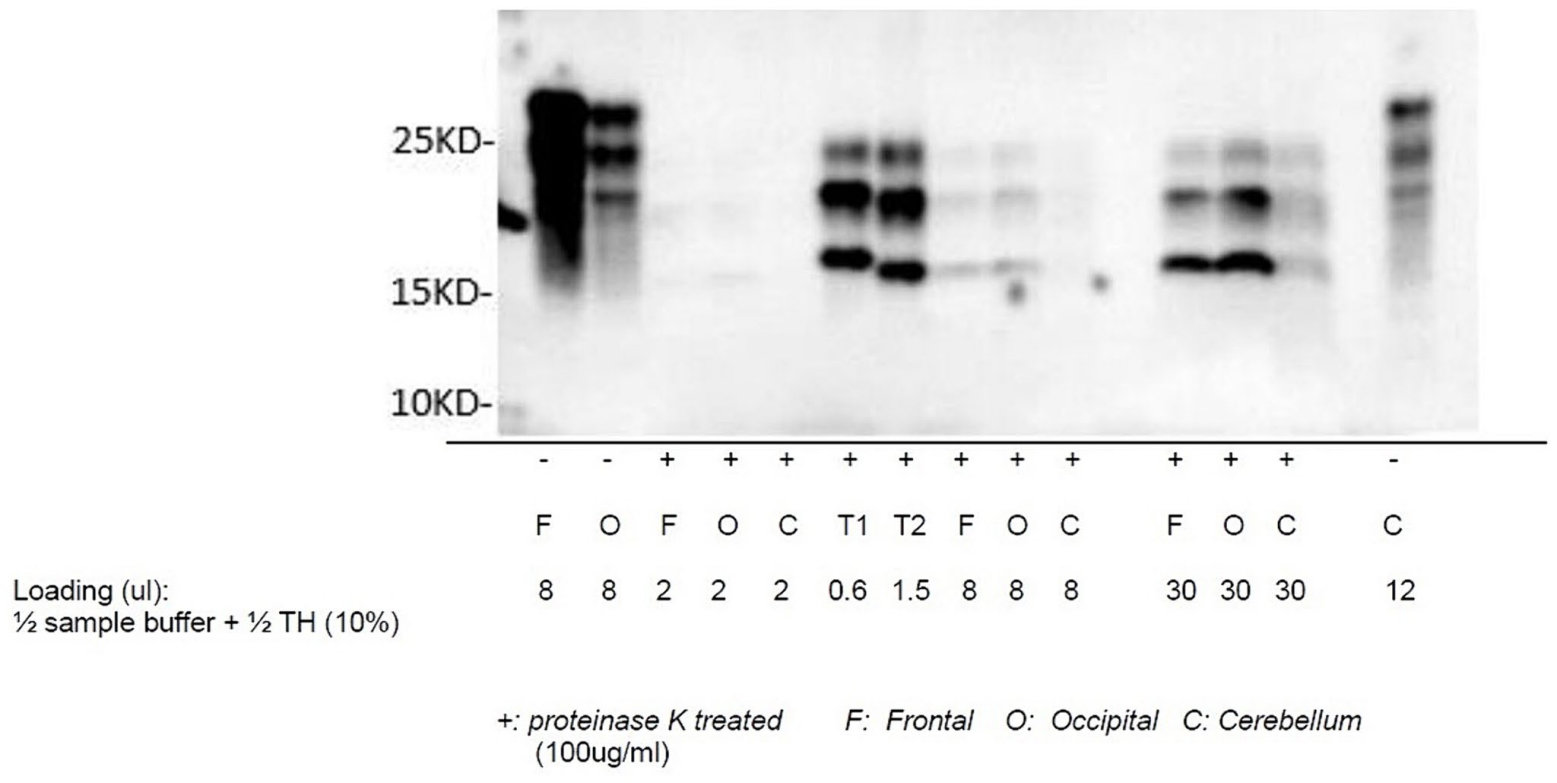
Western blot of sCJD: proteinase K-treated prion protein detected using antibody 3F4 utilizing samples collected from the frontal lobe, occipital lobe, and cerebellum. T1 and T2 are reference samples.

Autopsy was obtained. Western blot (Figure 2), hematoxylin plus eosin stain, and immunohistochemistry analysis (Figure 3) were positive for Sporadic Creutzfeldt-Jakob disease (MV1-2). Genetic analysis demonstrated no pathogenic mutations and an 129 M/V haplotype; 2 silent mutations were detected, c.351A > G and c.1-31G > A. The final diagnosis was sporadic Creutzfeldt-Jakob Disease MV1-2.

## Methods

### Eligibility criteria

All case studies related to Creutzfeldt-Jakob disease, initially diagnosed as a psychiatric disorder, were included in the systematic review. Studies that were written in a language other than English, unrelated neurology pathologies, cases that did not involve an initial psychiatric diagnosis, review articles, animal and *in-vivo* studies were excluded. The results of the search were categorized within Covidence, a web-based collaboration software platform that streamlines the production of systematic and other literature reviews. References were eliminated if they were duplicates and deemed not relevant in the screening and eligibility stage.

### Search strategy

A broad literature search of the PubMed database was completed on February 3, 2023 following the Preferred Reporting Items for Systematic Reviews and Meta-Analyses (PRISMA) checklist for studies and cases where Creutzfeldt-Jakob disease was initially diagnosed as a psychiatric disorder. Boolean search terms included “CJD” or “Creutzfeldt-Jakob disease” with three common psychiatric diagnoses, “Depression,” “Psychosis,” and “Mania”.

### Study selection

Two authors independently screened all studies, initially, by perusing titles and abstracts. Any relevant studies that were agreed upon would progress towards the full-text phase of the review. Full texts were reviewed according to the eligibility criteria as mentioned above. In case of disagreements, a third adjudicator was included to referee.

### Data extraction and analysis

In performing the systematic review according to our inclusion and exclusion criteria, two authors extracted a set of information from the full texts: age, gender, presenting symptoms, initial psychiatric diagnosis, whether patients underwent CT, MRI, PET, EEG, CSF studies, and the symptoms patients exhibited as they related to CJD.

## Results

**Figure 4:**
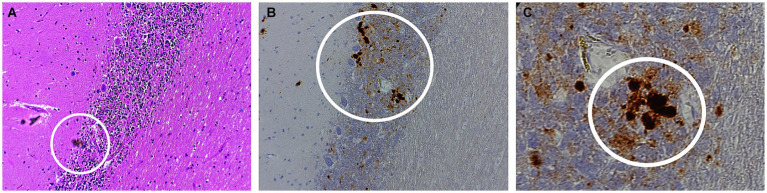
Histopathology of sporadic CJD MV1-2 **(A)** Hematoxylin–eosin (H&E) staining of the purkinje layers revealing kuru plaques **(B)** 10x and **(C)** 40x PrP immunostaining showing kuru histiotypic features. White circles denote the appearance of kuru plaques.

Utilizing the Boolean search strategy for all search terms, 393 articles were acquired from the PubMed registry and uploaded onto Covidence. Two-hundred and twenty one duplicate articles were removed. After titles and abstracts were screened, 123 articles were removed. All articles were able to be retrieved. Upon full text analysis for eligibility, 17 articles were excluded. Thirty-two articles remained with 43 total patients included in the systematic review with studies ranging from 1993 to 2021 (Figure 4).

Three common psychiatric diseases were evaluated as mimickers of CJD—depression, mania, and psychosis. Supplementary Table S1 outlines the initial diagnosis that were applied to each patient including basic demographics, past medical history, further progressive symptoms that helped to indicate that the symptoms may have a neurodegenerative rather than a psychiatric etiology, and diagnostics that helped to change the diagnosis from a psychiatric disorder to CJD. Progressive symptoms involve five of the most common signs and symptoms of CJD—neuropsychiatric symptoms such as dementia, behavioral issues, sleep disturbances, hallucinations, and frontal lobe signs (14–17), myoclonus particularly those of the startle variety (18), cerebellar manifestations such as nystagmus and ataxia (15, 18), signs of corticospinal tract (CST) involvement such as hyperreflexia, extensor plantar responses, and spasticity (19, 20), and other extrapyramidal signs sign as hypokinesia, bradykinesia, and rigidity.

Our review revealed 32 cases of CJD initially diagnosed as depression with two additional patients also receiving co-diagnosis of psychosis (21, 22). Patients were predominantly female (*n* = 26) with an average age of 58.3 years old (range 34–82, with one respondent described as middle age) (23). The percentage of patients who underwent electroencephalography (EEG), CT, MRI, PET, and cerebrospinal fluid (CSF) study were 91.4% (32/35), 25.7% (9/35), 88.6% (31/35), 5.7% (2/35), and 77.1% (27/35) respectively. Of the five signs and symptoms that convinced clinicians of an alternative diagnosis, neuropsychiatric symptoms were apparent in all patients (35/35), followed by 68.6% of patients (24/35) with extrapyramidal signs, 65.7% with cerebellar manifestations (23/35), 40% with myoclonus (14/35) and 34.3% with CST involvement (12/35).

Five cases were initially diagnosed as psychosis, with two additional patients also receiving the co-diagnosis of depression, resulted then CJD (21, 22). There was a slight male preponderance with an average age of 54.9 years old (range 31–82). The percentage of patients who underwent electroencephalography (EEG), CT, MRI, PET, and cerebrospinal fluid (CSF) study were 77.8% (7/9), 33.3% (3/9), 88.9% (8/9), 0% (0/9), and 66.7% (6/9) respectively. Of the five major CJD signs and symptoms, neuropsychiatric symptoms were apparent in all patients (9/9), followed by 66.7% of patients (6/9) with extrapyramidal signs, 44.4% (4/9) with cerebellar manifestations, 22.2% (2/9) with myoclonus, and 11.1% (1/9) with CST involvement.

Our review found one patient, a 45-year-old female, who was found to have symptoms of mania and was initially diagnosed with bipolar I disorder. She received EEG, MRI, and CSF studies and demonstrated persistence in all major CJD signs and symptoms.

While a multitude of diagnostic methods were used to resolve the CJD diagnosis of the 43 cases, they were used with varying degrees of success (Table 1). Positive EEG findings for CJD were seen on 26 of the 39 cases (66.7%) where EEGs were used. Positive MRI findings for CJD were seen in 30 of 37 cases (81.1%) where MRIs were used. Positive PET studies were seen in 1 of the 2 cases (50%) where PET imaging was used. Positive CSF studies, including 14-3-3, tau, and RT-QuIC were seen in 24 of the 33 cases (72.7%) that were used as diagnosis. While most patients were stratified according to the WHO classification of CJD based on clinical findings and imaging, fourteen patients underwent neuropathologic studies, including immunohistochemistry, western blotting, and genetic testing to definitively confirm the diagnosis (1, 18, 22, 24–33).

**Table 1:** A diagrammatic summary of all cases that were acquired in the systematic review, including gender and age demographics.

Total number of studies	43	
Females	26	
Average age	58.3	
Total EEG tests completed	39	
Positive EEG findings		26 (66.7%)
Total CT imaging completed	12	
Positive CT findings		1 (8.3%)
Total MRI completed	37	
Positive MRI findings		30 (81.1%)
Total PET completed	2	
Positive PET findings		1 (50%)
Total CSF studies completed	33	
Positive CSF studies		24 (72.7%)

Positive findings in EEG, CT, MRI, and CSF studies are shown as a percentage of the total number of times that the diagnostic was used. Positive CSF studies include 14-3-3, tau, and RT-QuIC abnormalities.

## Discussion

The diagnosis of CJD starts from an accurate assessment of clinical presentation. In conjunction with World Health Organization (WHO) Criteria for Diagnosis of Sporadic CJD, Hermann et al., provides a succinct understanding of possible, probably, and definite diagnosis for CJD, updated for the addition of RT-QuIC diagnosis modality (34, 35). While most studies in our cohort achieve the criteria for probable and possible sporadic CJD based on neurological manifestations with relevant laboratory tests, only a few underwent biopsies to achieve the diagnosis of definite CJD. The difficulty in identifying possible CJD and subsequent misdiagnosis as a psychiatric disorder is owed to its wide phenotypic variance between cases. As seen in our patient and in Bartlett et al., prion infectivity is not confined to neuronal tissue as both patients demonstrated chest pain, an underreported feature of the disease (36). The scrapie isoform of the prion protein (PrP^Sc^) has been found in skeletal and cardiac muscle and may lead to extra-neural pathology before the neurologic and psychiatric symptoms have manifested (20, 37–39).

Our review focused on the numerous paraclinical tests that were used to support or refute the diagnosis of CJD, with varying levels of success. Concurrent and even multiple psychiatric symptoms and diagnoses can make CJD diagnosis a difficult undertaking. Abudy et al. describes how one paraclinical test may have to be utilized more than once to achieve a positive result. Even with the most popular modality, MRI brain, difficulties or uncertainties with imaging interpretation may confound an accurate diagnosis, as was seen with our case presentation. Several patients in our cohort required multiple MRI scans in order to arrive at the correct diagnosis (22–27, 29, 32, 40–43). Our review also demonstrates the relative ineffectiveness of certain tests. CT head was documented to be used in several patients in our cohort; however, it should not be used for diagnosis of CJD since its imaging findings are neither sensitive nor specific (44), although its utilization can help rule out other diseases and help physicians boost their differential diagnosis approach (45). Per our systematic review, eleven out of the twelve that documented the use of CT scans revealed negative results.

Several types of CSF studies have been used over the years to help diagnose CJD. Tau and 14-3-3 were the most common tests of choice for earlier studies. In recent years, RT-QuIC has became the CSF diagnostic test of choice for prion disease. Per our analysis, three patients underwent RT-QuIC for diagnosis of CJD (23, 46–48). RT-QuIC was positive in two patients (23, 47) and negative in one patient (48). The seemingly low sensitivity rate of RT-QuIC is most likely due to low power (*n* = 3). In total, 33 patients underwent CSF studies with a false negative rate of 27% (9/33). The unusually high rate of false negative is most likely due to (1) selection and publication bias where only biopsy and autopsy revealed prion disease when all other tests claimed the opposite and (2) older cases did not use RT-QuIC as a diagnostic test.

### Limitations

Several limitations were encountered during the study. Firstly, while a neurological and psychological assessment was acquired from the patient in the inpatient setting, a comprehensive neuropsychological assessment was not possible. The patient’s medical course was too unstable for a neuropsychological assessment to be attempted in the inpatient setting. Future studies can be set up such that suspected CJD cases could be scheduled for neuropsychological testing as soon as suspected. However, the clinical utility of an in-depth neuropsychological evaluation may be debatable in this setting, save for slow-progressing cases. Rather we stress the utility of a proper and comprehensive neurological examination, which can be prompt and logistically flexible. Secondly, our systematic review of papers from 1993 to 2021 uncovered a broad spectrum of methods that authors used to arrive at the diagnosis of CJD. Many of the earlier studies utilized biopsies and autopsies to help diagnose CJD (26, 27, 32, 33). Later studies took advantage of the physical exam, diagnostic studies such as EEG, MRI, and CSF studies and stratified the probability of CJD according to the WHO criteria (20, 40, 47, 49). While immunohistochemistry and western blot are now commonly used in practice, we found many studies utilized merely a positive 14-3-3 and tau in arriving at the diagnosis of CJD. Additionally, cases such as those by Dervaux et al., and Azorin et al., demonstrated that patients can have positive CJD diagnosis via pathological samples despite EEG, MRI, and CSF studies being negative. Thirdly, due to its phenotypical variances, CJD is a difficult disease to diagnose, and, despite our best efforts, we may not have acquired all cases of CJD that were initially diagnosed as psychiatric disorders.

## Conclusion

The present case and our systematic review illustrate multiple patients with CJD initially diagnosed with psychiatric disorders such as depression, bipolar disorder, and psychosis. Our review suggests that CJD should be considered in patients who initially present with psychiatric symptoms, with abnormalities on brain MRI, poor response to treatment and progression to further cognitive difficulties. Early recognition can assist with goals of care conversation, potentially limiting healthcare costs.
